# Stability and equation of state of face-centered cubic and hexagonal close packed phases of argon under pressure

**DOI:** 10.1038/s41598-021-93995-y

**Published:** 2021-07-26

**Authors:** Agnès Dewaele, Angelika D. Rosa, Nicolas Guignot, Denis Andrault, João Elias F. S. Rodrigues, Gaston Garbarino

**Affiliations:** 1 CEA, DAM, DIF, 91297 Arpajon, France; 2 Université Paris-Saclay, CEA, Laboratoire Matière en Conditions Extrêmes, 91680 Bruyères-le-Châtel, France; 3 ESRF, BP220, 38043 Grenoble, France; 4 Synchrotron Soleil, 91192 Saint Aubin, France; 5 Université Clermont Auvergne, CNRS, IRD, OPGC, LMV, Clermont-Ferrand, France

**Keywords:** Condensed-matter physics, Phase transitions and critical phenomena, Structure of solids and liquids

## Abstract

The compression of argon is measured between 10 K and 296 K up to 20 GPa and and up to 114 GPa at 296 K in diamond anvil cells. Three samples conditioning are used: (1) single crystal sample directly compressed between the anvils, (2) powder sample directly compressed between the anvils, (3) single crystal sample compressed in a pressure medium. A partial transformation of the face-centered cubic (fcc) phase to a hexagonal close-packed (hcp) structure is observed above 4.2–13 GPa. Hcp phase forms through stacking faults in fcc-Ar and its amount depends on pressurizing conditions and starting fcc-Ar microstructure. The quasi-hydrostatic equation of state of the fcc phase is well described by a quasi-harmonic Mie–Grüneisen–Debye formalism, with the following 0 K parameters for Rydberg-Vinet equation: $V_0$ = 38.0 Å$^3$/at, $K_0$ = 2.65 GPa, $K'_0$ = 7.423. Under the current experimental conditions, non-hydrostaticity affects measured *P*–*V* points mostly at moderate pressure ($\le$ 20 GPa).

## Introduction

Argon (*Z* = 18) is the most abundant rare gas in the Earth’s atmosphere, in accordance with accretion models^1^. Because of this relative abundance and its inertness, it is widely used for storage or treatment of reactive materials. In high pressure technologies, argon is a common pressure transmitting medium, which can be loaded in diamond anvil cells either cryogenically or under pressure.

Compressed rare gases are also ideal test cases for theoretical chemistry methods, because of the low interaction energy between atoms and the fast convergence of the many-body expansion of the interaction energy. Already in the 1980’s, high-pressure equation of state and melting curve of fcc argon have been reproduced and extended with a good accuracy using simple pair potentials, coupled with Monte-Carlo simulations^2–4^. Recent measurements of argon equation of state, using modern synchrotron techniques, focused on the very high pressure domain ($P \ge$ 40 GPa)^5,6^. The prediction of the structure adopted by rare gas solids is more difficult to address, because face-centered cubic and hexagonal close packed rare gas solids are almost isoenergetic, so that subtle effects such as many-body interactions, or phonon dispersion curves play a role in their relative stability^7–9^. Rare gases crystallize from the liquid under fcc structure, and it has been observed that compression favors the hcp phase in the case of xenon/krypton, with a sluggish transition observed or expected around 80 GPa/400 GPa at 300 K^10–12^. Hcp argon has been observed as a minor phase in coexistence with fcc above 50 GPa after heat treatment^5^, and was expected to become the major phase around 300 GPa. However, it could not be detected after cold compression up to 248 GPa^6^, which suggests that the fcc to hcp transition might take place under even higher pressures.

The motivation of this work is to provide reference equation of state data for argon in the 0–120 GPa range, suitable for high pressure metrology purpose and testing the accuracy of various numerical calculations, and to better understand the onset of the fcc-hcp transition in that compression range. At ambient temperature, fcc argon crystallizes at 1.4 GPa^13^, and shows evidence of nonhydrostatic behavior at pressures as low as 2 GPa^14^. It becomes even stronger above 30 GPa, with pressure gradients of more than 1 GPa/10 $\upmu$m^14,15^; this might have biased equation of state data measured by compressing argon samples, which completely fills the high pressure chamber of diamond anvil cells^14,16–20^. Here, we used pure argon but also grew argon crystals embedded in an ArNe$_2$ laves phase matrix, which acts as a soft pressure transmitting medium around it^21^, enabling an accurate equation of state measurement as detailed below. We used state-of-the-art synchrotron techniques, which allows detecting minor amounts of hcp phase appearing during the compression of solid argon and to better constrain the mechanism of the fcc-hcp phase transformation for this rare gas solid. To finish, we collected many data at low pressure from room to low temperature to test the accuracy of a quasi-harmonic equation of state and constrain tightly its parameters.

## Results

Several samples have been loaded in membrane diamond-anvil cells with rhenium gaskets (see Table 1). In runs 2 and 3, Ar-Ne mixtures have been studied; runs 1, 4 and 5 samples were pure argon. They have been characterized with X-ray diffraction (XRD) on different platforms and under pressure-temperature conditions summarized in Table 1. Argon samples exhibited different textures in different runs.

### Sample textures

#### Runs 1 and 4

**Figure 1:**
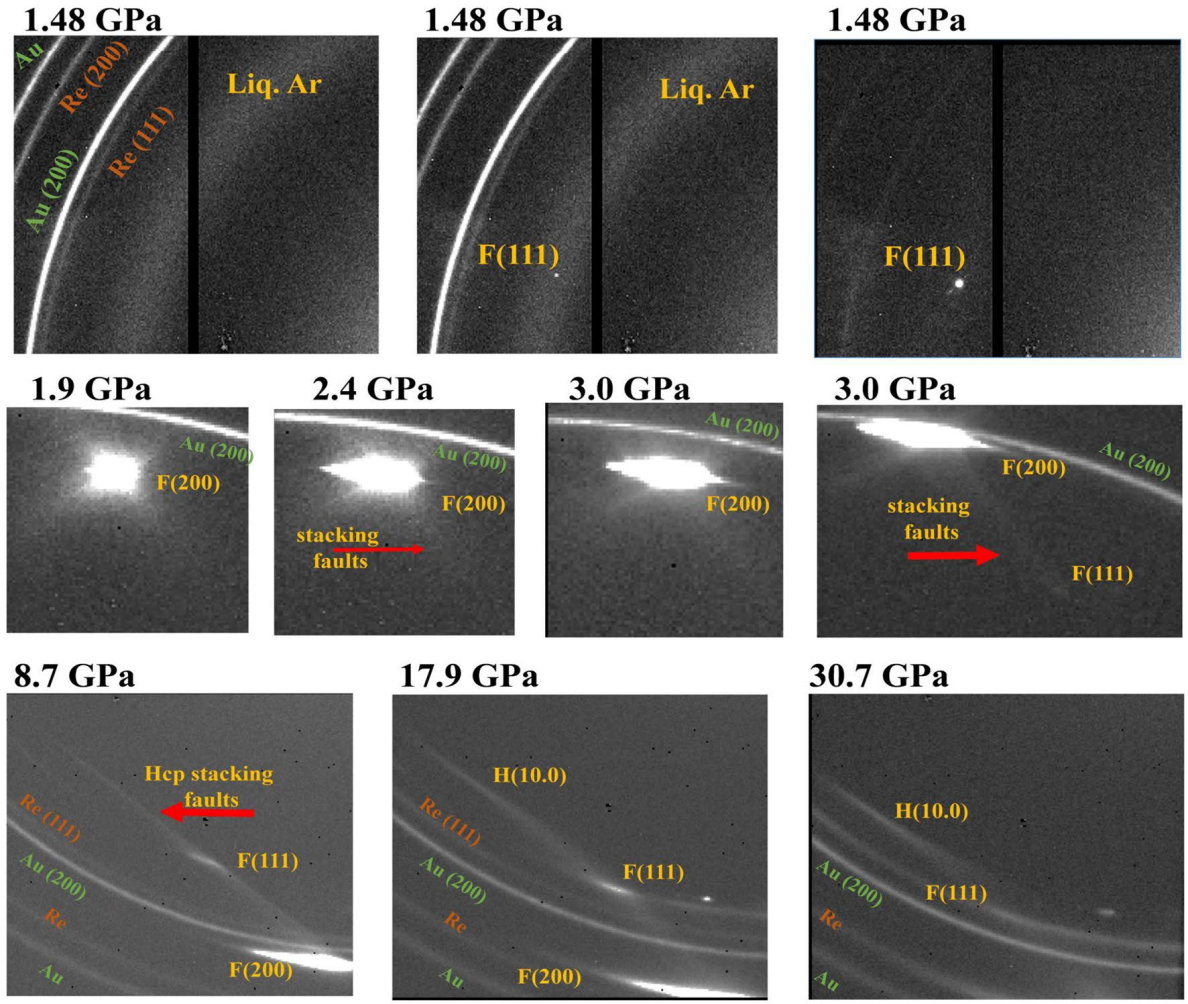
Textures in pure Ar from bidimensional X-ray diffraction images collected in run 1. At 1.48 GPa, Ar grows as a few single crystals from the melt. Evidences of non-hydrostatic compression—a broadening of single crystal XRD peaks—appear around 2.4 GPa. Stacking faults give rise to a structured diffuse signal above 1.9 GPa, which progressively transforms into (100) (labelled H(10.0)) and (101) hcp XRD peaks.

Pure argon samples studied at 296 K (runs 1 and 4) crystallized at $\sim$1.4 GPa, in good agreement with the measurement of Ref.^13^, under the form of a single crystal (see Fig. 1). Then, membrane pressure was smoothly increased so that one single crystal completely filled the sample cavity; one pattern containing both the gold pressure standard and argon XRD signal was collected at each pressure step up to 23 GPa; above 23 GPa, two XRD spectra were collected, one on Ar and one on Au+Ar. The zone analyzed with X-ray consisted of one single crystal which orientation could be determined from XRD. Remarkably, dense planes were almost parallel to the diamond anvil surface. These planes were (100) and (112) for runs 1 and 4, respectively. This is probably due to the ratio between lattice parameters of Ar and diamond at 1.4 GPa ($a_C \sqrt{2} \sim a_{Ar}$), which favors an epitaxial growth of argon on the (100) surface of diamond anvil. In Fig. 1, diffuse streaks can be seen around single crystal fcc Ar XRD spots. Classical interpretation of these features is stacking faults^22^, which are indeed common crystallographic defects in fcc crystals.

#### Runs 2 and 3

**Figure 2:**
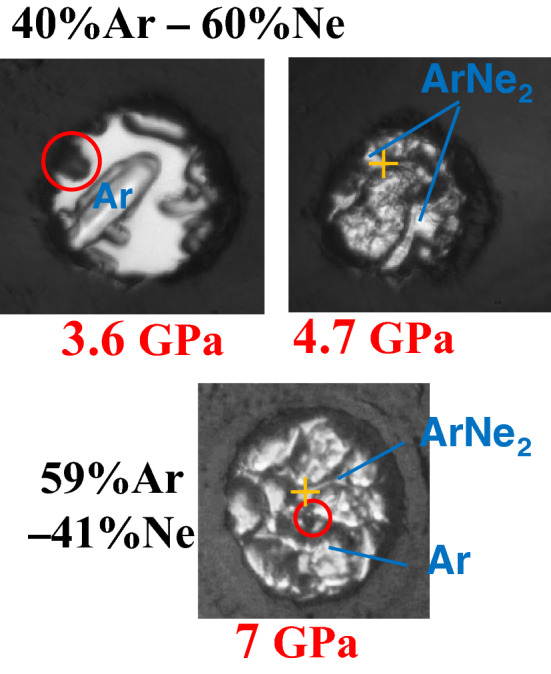
pictures of the sample cavities for runs 2 and 3. The two pictures for run 3 have been taken on pressure increase. Gold pressure marker is circled in red, and yellow crosses indicate the position of XRD beam where most of exposures have been performed. It is in ArNe$_2$, at the edge of an argon crystal.

In the mixture samples (runs 2 and 3), two solid phases were observed, as expected from the Ar–Ne binary diagram presented in Ref.^21^. An argon-rich single crystal appeared in the fluid phase at 2.5 GPa and 3.5 GPa in run 2 and run 3, respectively (see Fig. 2); the fraction of solid phase increased up to 4.7 GPa, at which the remaining fluid crystallized to form a solid identified in Ref.^21^ as an ArNe$_2$ Laves phase with a MgZn$_2$ structure. Thus, above 4.7 GPa samples consisted of Ar-rich single crystals, embedded in ArNe$_2$ matrix.

It is important to know whether the Ar-rich solid studied in runs 2 and 3 was pure argon, or argon with neon impurities. Ref.^21^ reports that in the Ar–Ne mixture with the highest argon content, 92%, a solid–solid demixtion was observed at 5 GPa which places an upper bound of solubility of neon in argon under pressure: 8%. Measured volumes of argon and argon-rich solid under same conditions allow to better constrain this solubility. The volumes appear to be identical around 4 GPa, within experimental uncertainty, 0.1%. From pure argon and pure neon^23^ volumes obtained at 4 GPa, we estimate the solubility of neon into solid argon to be of the order of 0.2 mol% at maximum. We thus make the assumption that the amount of neon incorporated into the Ar-rich solid is low enough to have little effect on its behaviour under high pressure.

**Figure 3:**
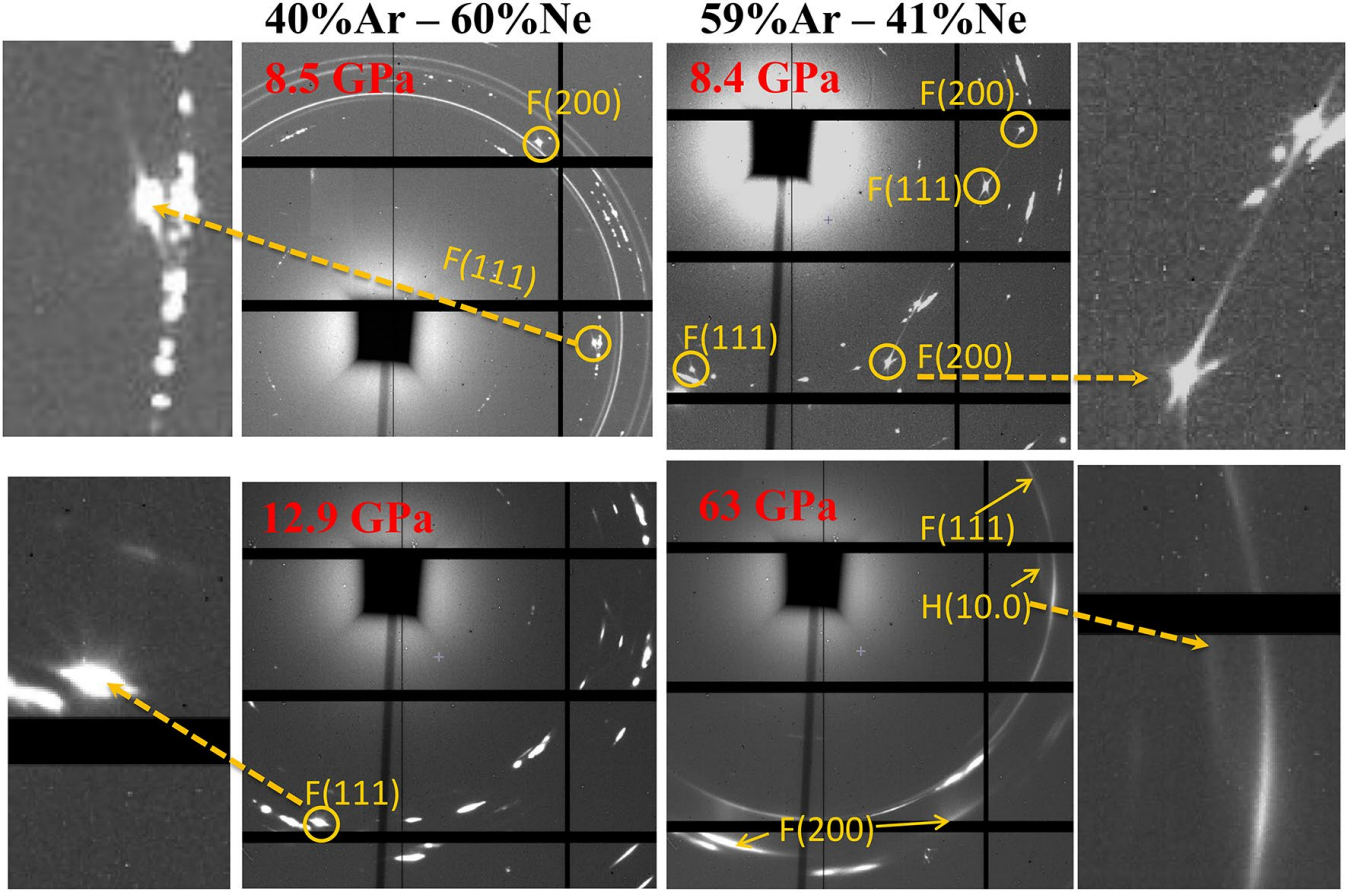
Textures in Ar-Ne mixtures (runs 2–3). Portions of bidimensional X-ray diffraction patterns collected in Ar–Ne mixtures (uppper left corner: run 3; others: run 2). The square shadow of the beamstop marks the position of pattern center. Ar XRD peaks are labelled in yellow. The remaining peaks correspond to ArNe$_2$ for run 2, and ArNe$_2$ and gold (continuous ring) for run 3. Evidence of non-hydrostatic compression—a broadening of Ar single crystal XRD peaks—appears above 10 GPa in run 2. Around 8.5 GPa, stacking faults create a structured diffuse signal around single crystal Ar XRD spots for both samples, which clearly appears in the enlarged pictures. This signal progressively transforms into (100) (labelled H(10.0)) hcp XRD peak.

In order to measure argon crystals which would not be directly compressed between the diamond anvils, we placed the X-ray beam at the edge between argon crystal and ArNe$_2$ matrix; the Au pressure marker is located inside ArNe$_2$ (Fig. 2). Figure 3 represents XRD images collected in runs 2 and 3: streaks around Ar-rich single crystal XRD spots similar to those observed in run 1 can be noticed, showing the formation of stacking faults.

#### Run 5

**Figure 4:**
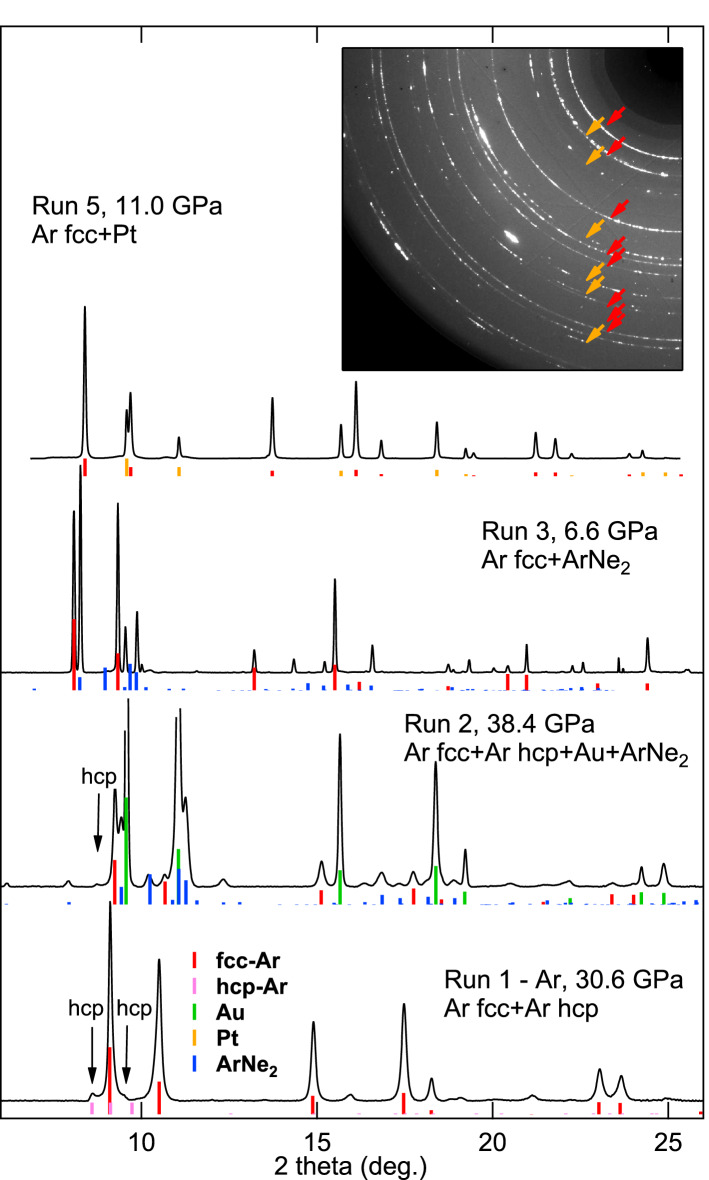
XRD patterns collected for four experimental runs at various pressures. Tick marks indicate the positions and predicted intensities for perfect powders of each compound in the pressure chamber; the arrows indicate the positions of diffraction peaks for hcp-argon. The inset represents the XRD image corresponding to run 5 pattern. In this run, argon sample was a fine powder and XRD rings are identified with red and orange arrows, corresponding to fcc Ar and platinum, respectively.

In cryogenic run 5, we have followed the inverse strategy to runs 1–4: the starting single crystal(s) have been laser-heated using a platinum absorber before placing the diamond anvil cell in the cryostat to obtain a powder with sub-micrometer-sized cristallites. This treatment enabled standard powder analysis of argon XRD signal. One typical XRD image collected in run 5 is presented in the inset of Fig. 4.

#### Analysis of XRD images

We have used various strategies to analyze XRD data, adapted to samples textures. XRD signal from argon samples collected in runs 1 and 2 was of sufficient quality to enable a Rietvelt refinement with a texture determination. The softwares Dioptas^24^ or Fit2D^25^ were used to generate slices of 1-dimensional diffraction patterns along the azimuth suitable for Rietvelt refinement of textured samples (see Fig. 4, Fig. S1). MAUD software was employed to extract the unit cell volumes, volume fractions and crystal domain sizes for runs 1 and 2. These quantities were obtained by adjusting the XRD Bragg reflection profiles using Popa’s analytical approximation^26^. For run 3, the amount of Ar was too low compared to ArNe$_2$ to allow such treatment and unit cell volumes were determined by XRD peaks fitting. Run 4 sample exhibited a high plastic strain and no texture refinement was performed, unit cell volumes were determined by XRD peaks fitting. For run 5, the data indicated no texturing of argon samples so that standard Rietvelt analysis of integrated patterns has been performed with MAUD.

### Non-hydrostatic stress in sample cavities

In the literature, cases where non-hydrostatic stress affect the measured EoS have been discussed, such as systematic bias^16,19,20^, unphysical lattice distortions^17,18^. This stress should thus be evaluated here in order to discuss argon EoS.

Ar XRD signal collected on image plates witnesses the effect of non-hydrostatic compression on Ar texture. In Fig. 1, the round single crystal (111) Ar XRD peak collected at 1.48 GPa clearly broadens above 2.4 GPa, along the azimuthal angle. It progressively transforms into a textured powder XRD ring at 17.9 GPa: the single crystal, directly compressed between the diamond anvils, undergoes severe plastic strain. This is due to the decrease of the gap between diamond anvils with increasing pressure (by at least a factor of two^27^). The same happens in run 2 (Ar embedded in ArNe$_2$ matrix), but to a lesser extent because softer ArNe$_2$ also strains to adapt to this sample geometry change. In Fig. 3, an azimuthal broadening is noticeable for Ar (111) peak at 12.9 GPa in run 2. The shape of Ar XRD peaks are similar at 12.9 GPa for run 2 and 2.4 GPa for run 1, evidencing a lower plastic strain in run 2.

**Figure 5:**
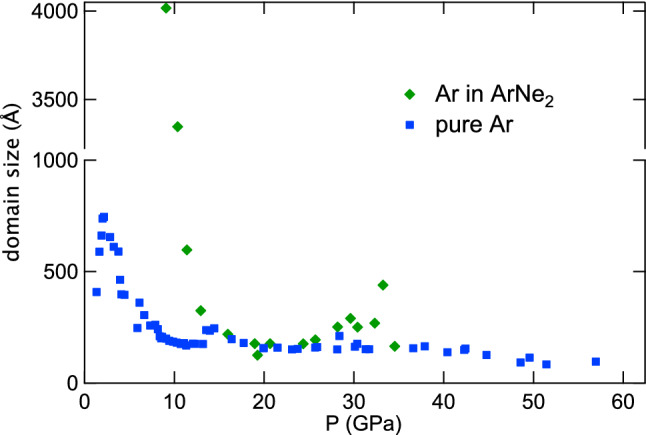
Size of fcc-Ar domains obtained from Rietvelt refinement of XRD data for runs 1 (pure Ar) and 2 (Ar in ArNe$_2$). The vertical axis is split.

The Rietvelt analysis^26^ of XRD images yields an estimate of crystal domains size in the sample, providing another insight into its texture. In Fig. 5, the domain size in run 1 (pure argon) and run 2 are represented vs pressure. For run 2, an overlap between Au and Ar XRD lines hindered this treatment above 35 GPa. For pure argon, domain size decreases above 2 GPa, to reach a minimum value of $\sim$170 Å around 10 GPa. The same happens in Ar compressed in ArNe$_2$, but at higher pressure, the minimum being reached around 20 GPa.

Both analyses suggest that non-hydrostatic pressurization induces plastic strain in Ar, in all samples, but at $\sim$ 10 GPa higher pressure for Ar embedded in ArNe$_2$ than in pure Ar.

**Figure 6:**
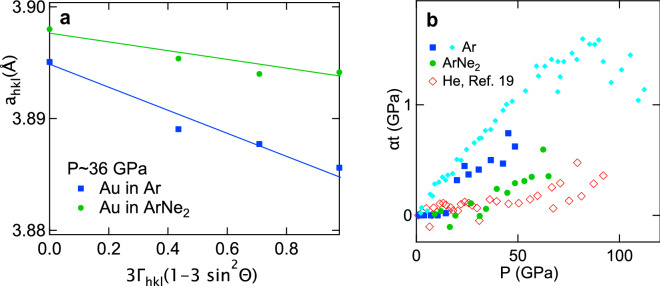
Evaluation of non-hydrostatic stress in runs 1 and 2 based on Au X-ray diffraction lines position. (**a**) Gamma plot for Au around 36 GPa. In this plot and underlying model, the slope of $a_{hkl}$ vs $3 \times \Gamma _{hkl} \times (1-\sin ^2 \theta )$ is proportional to uniaxial stress *t* in Au. (**b**) $\alpha t$ ($\alpha \sim$ 0.5) estimated with this analysis, vs pressure, in runs 1, 2 and 4. It is compared to values estimated for Au compressed in He^19^.

A standard analysis of gold diffraction lines has been performed to evaluate the stress on this material and thus pressurizing conditions in pure Ar and in ArNe$_2$. The principle is the following: under non-hydrostatic compression, the XRD peaks measured in a conventional DAC geometry are shifted from those measured under hydrostatic compression, by an amount which depends on the peak class^19,28^. The “gamma-plots” in Fig. 6 represent the cubic lattice parameter corresponding to each XRD line ($a_{\mathrm {hkl}}=d_{\mathrm {hkl}} \times \sqrt{(}h^2+k^2+l^2)$), as a function of diffraction angle 2$\theta$ and $\Gamma _{hkl}=(h^2k^2+h^2l^2+k^2l^2)/(h^2+k^2+l^2)^2$. In short, it shows the deviation of the XRD lines from the cubic symmetry, due to non-hydrostatic stress. The model and notations are presented in Ref.^19,28^. Under non-hydrostatic compression, this plot shows a linear trend with a slope *M* proportional to the deviatoric stress $t=\sigma _3-\sigma _1$ ($\sigma _3$/$\sigma _1$ vertical or compression axis/horizontal stress):

1 $$\alpha t \simeq - \frac{{3 \times M}}{{a_{{111}} \times S}},$$

With *S* the elastic anisotropy parameter defined and estimated for gold in^19,28^. $\alpha$ value is usually estimated to 0.5.

The gamma-plots represented in Fig. 6a exhibit a linear negative trend for Ar and ArNe$_2$ pressure transmitting media, as expected and with a stress along compression axis $\sigma _3$ higher than the horizontal stress $\sigma _1$. The product $\alpha t$ estimated using the same gold elastic anisotropy as in Ref.^28^ is plotted in Fig. 6b; error bars are $\sim$ 0.3 GPa. Comparison with a literature study^19^ shows that $\alpha t$ has a similar value in Ar/ArNe$_2$/He at 20 GPa/50 GPa/80 GPa. The amount of non-hydrostatic stress sustained by light rare-gas solids would thus increase with *Z* from helium to argon, a conclusion in line with Ref.^14^. Compressing Ar in ArNe$_2$ should thus reduce non-hydrostatic compression effects on the measured EoS, compared to compression of pure argon.

### Equation of state of face centered cubic Argon

#### Quasi-hydrostatic Fcc-Ar EoS parameters

**Figure 7:**
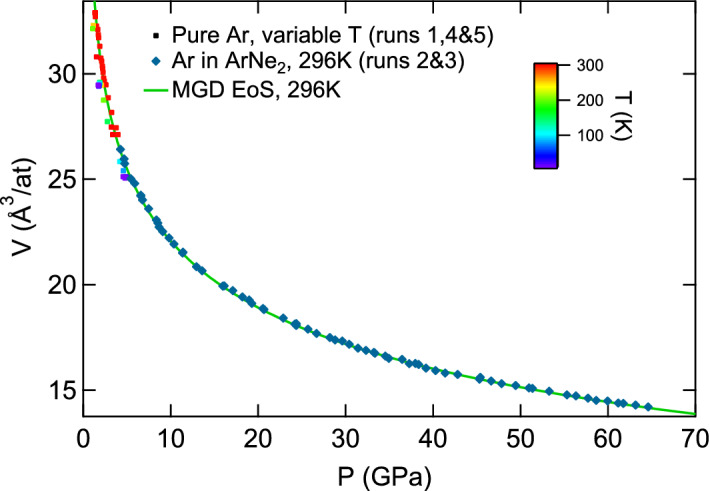
Quasi-hydrostatic equation of state of fcc-Ar. Mie–Grüneisen–Debye (MGD) EoS is described in the text and Table 2.

In order to minimize the bias due to non-hydrostatic compression on EoS parameters, we have used the data collected for argon embedded in ArNe$_2$ as a reference for fcc-Argon equation of state, together with data collected for pure argon at $P \le$ 5 GPa to constraint low pressure volume (see Fig. 7). The data points are provided as a supplementary file.

In this study, a majority of XRD data have been collected at 296 K; usually, isothermal compression curves can be fitted with isothermal EoS formulations, such as Rydberg-Vinet^29^, with volume and bulk modulus of the solid under ambient conditions as fit parameters. However, argon is gaseous under ambient conditions and Rydberg-Vinet formalism cannot be used at 296 K for this element. Following Finger et al.^2^, we express the pressure in solid fcc argon as a sum of a static pressure $P_0(V)$ (expressed with a Rydberg–Vinet equation of state, $V_0$ being the equilibrium volume at ambient pressure and 0 K) at 0 K and a thermal pressure $P_{th}(V,T)$ estimated with the quasi-harmonic Mie–Grüneisen Debye (MGD) formalism: $P(V,T)=P_0(V)+ P_{th}(V,T)$, with ($x=V/V_0$):

2 $$\begin{aligned} P_0(V)=3 K_0 (1-x^{1/3}) x^{-2/3} \exp \left( \frac{3}{2} (K'_0-1)(1-x^{1/3})\right) , \end{aligned}$$

and

3 $$\begin{aligned} P_{th}(V,T) = \frac{3\gamma R T}{V} \left[ D \left( \frac{\theta _D}{T}\right) \right] \end{aligned}$$

(zero point pressure is neglected). In Eq. (2), the parameters $K_0$ and $K'_0$ are the bulk modulus and its pressure derivative at ambient pressure and 0 K. In Eq. (3), *D* is Debye function, $\theta _D$ Debye temperature and $\gamma$ Grüneisen parameter ($\gamma =-\partial \ln \theta _D / \partial \ln V$). $\gamma$ is decreasing with compression^30^; to prevent overfitting, we assumed the following behavior:

4 $$\begin{aligned} \gamma (V) = 2.20 \times x +0.5. \end{aligned}$$

This behavior has been suggested in Ref.^2^ on the basis of measured Grüneisen parameter under ambient pressure and low temperature, and assuming a limit value of 0.5 under infinite compression. Debye temperature then expresses as:

5 $$\begin{aligned} \theta _D(V) = 93.3 \sqrt{(}1/x) \times \exp (2.20(1-x)), \end{aligned}$$

93.3 K being its value under low T and ambient pressure.

We have fixed $K_0$ to the low temperature value measured in Refs.^31,32^: $K_0=2.65$ GPa. The parameters $V_0$ and $K'_0$ have been obtained by a fit of the 300 K data points represented in Fig. 7: $V_0$ = 38.0 Å$^3$/at and $K'_0$ = 7.423. $V_0$ is close to the value measured in Refs.^31,32^, 37.45 Å$^3$/at. The parameters of quasi-hydrostatic Mie–Grüneisen–Debye (MGD) EoS are listed in Table 2.

**Figure 8:**
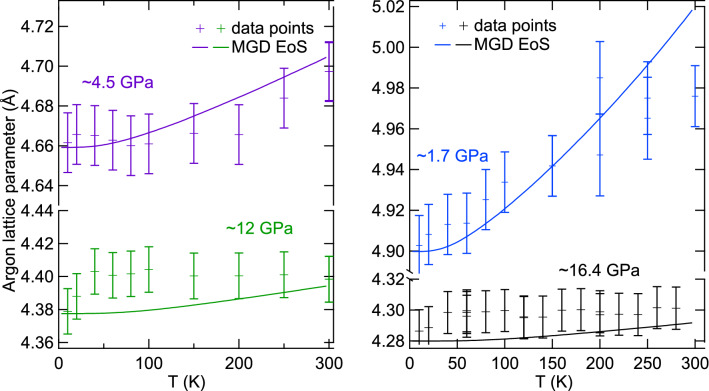
Thermal expansion of fcc-Ar. Argon lattice parameter has been measured vs temperature along four isobars at $\sim$ 1.7 GPa, $\sim$ 4.5 GPa, $\sim$ 12 GPa and $\sim$ 16.5 GPa. The crosses are data points, slightly modified to correct measured pressure variations (± 0.5 GPa). Continuous lines are the Mie–Grüneisen–Debye (MGD) model described in the text and Table 2.

We have tested these parameters using low temperature high accuracy data collected in run 5. Figure 8 represents two isobars collected below ambient temperature, compared with MGD EoS. Thermal expansion of argon is clear at 1.7 GPa and decreases with compression, so that it almost vanishes above 16 GPa; this is well reproduced by the MGD EoS. This validates the effect of compression on $\gamma$—which is proportional to thermal expansion—assumed in Eq. (4).

It is expected that argon behaves as a quasi-harmonic solid out of the temperature range scanned here, as neon^23^. The current MGD EoS could thus be used for pressure calibration purpose when argon is used as a pressure transmitting medium at ambient temperature, but also in resistively or laser heated diamond anvil cell experiments. For these experiments, an ideal pressure gauge would have a vanishing thermal expansion (or thermal pressure), so that measured volume gives directly the pressure. The current argon EoS predicts that the thermal pressure reaches $\sim$ 10 GPa at 3000 K and 100 GPa, to be compared with $\sim$ 15 GPa for neon^23^ and $\sim$ 6.5 GPa for KCl or KBr^33^. KCl and KBr thus appear as better pressure gauges for laser-heating experiments than argon (and neon) because the pressure estimated using their EoS will be less affected by temperature uncertainties.

#### Fcc-Ar compression measured in pure argon

**Figure 9:**
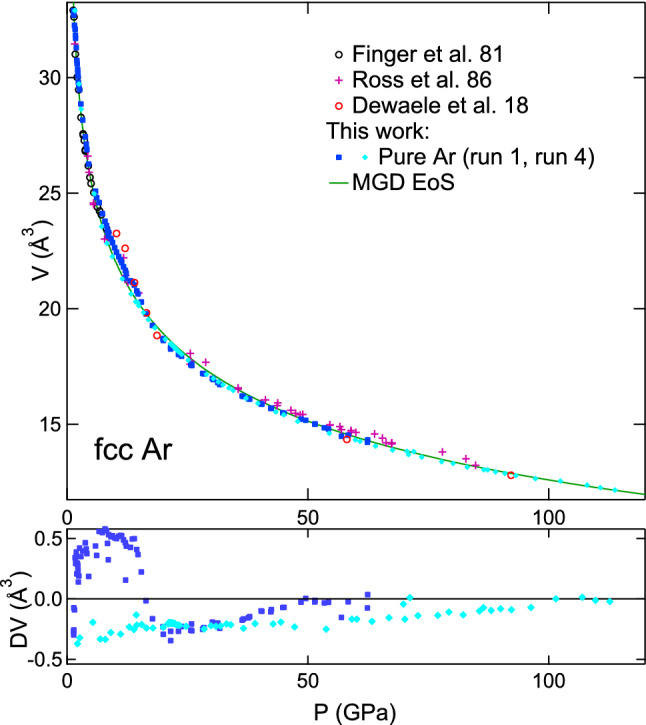
Equation of state of fcc-Ar directly compressed between diamond anvils. Top graph: atomic volume vs pressure measured with Au standard^28^. Current and literature^2,3,6^ experimental *P*–*V* points have been plotted, with an updated pressure metrology for Refs.^2,3^. Bottom graph: difference between measured volume and volume calculated using quasi-hydrostatic Mie–Grüneisen–Debye (MGD) EoS described in the text and Table 2.

Argon *P*–*V* points measured under non-hydrostatic compression up to 114 GPa are compared to quasi-hydrostatic EoS in Fig. 9. The difference is smaller than 2%, and the relative difference becomes very small under high compression. This could be due to cupping of the diamond anvils, which is expected to decrease non-hydrostaticity; indeed, $\alpha t$ estimated from gold XRD lines is plateauing above 70 GPa (Fig. 6). In Dewaele et al.^6^, the EoS of argon compressed in a toroidal diamond anvil cell was measured up to 248 GPa, using the XRD signal of rhenium gasket to estimate the pressure. The *P*–*V* points get close to the current ones above 100 GPa, after a first compression stage which produces a different stress distribution in this device and a systematic deviation from the quasi-hydrostatic EoS^6^.

The volume measured in run 1 exhibits a sharp decrease around 16 GPa—4% in 3 GPa, see the lower panel of Fig. 9, but not in run 4 where conditions were different (different size of culet and sample chamber, X-ray spot size, and location of X-ray gauge, Table 1). This suggests that the stress reorganized in run 1 sample around 16 GPa producing this jump in measured *P*–*V* points, possibly due to gasket flow or to the appearance of the hcp phase (see below). In fact, anomalies of the compression curve have been linked to the fcc-hcp phase transformation in heavy rare-gas solids (krypton and xenon)^11,12^. However, in Refs.^11,12^, the anomaly appeared when the sample contained 20 % vol. of hcp phase, an amount which is not reached here (see below). It can be noted that a 4% anomaly measured under non-hydrostatic stress in Zr compression curve, first interpreted as an evidence of an isostructural phase transformation, was not confirmed when the sample was compressed in a soft pressure transmitting medium^20^. This shows that detailed features of EoS collected under non-hydrostatic compression should be interpret with caution.

### Hexagonal close packed argon

The hcp phase of argon has been observed in runs 1–4, but not in cryostat experiments (run 5) performed up to 16 GPa. In runs 1–4, samples were single crystals which made the observation of minor amounts of hcp modification easier, as shown in Figs. 1 and 3. In run 3 (lowest argon content), hcp (100) peaks appears around 35 GPa but remained barely noticeable and could not be analyzed quantitatively. In run 1 and 2, several hcp XRD peaks can be followed above 4.6 GPa and 13 GPa, respectively. Figure 1 shows how streaks caused by stacking faults in fcc crystal continuously evolve to form a hcp (100) peak, indicating a link between these defects and the fcc $\rightarrow$ hcp transformation, thus producing a highly textured hcp phase. The same was observed in runs 2 and 3 samples. It is interesting to note that the size of fcc-Ar domains plotted in Fig. 5 decreases below 500 Å at the same time as hcp signal appears in both samples. Unfortunately, the low hcp signal prevented a measurement of the crystallographic orientation of hcp cristallites.

**Figure 10:**
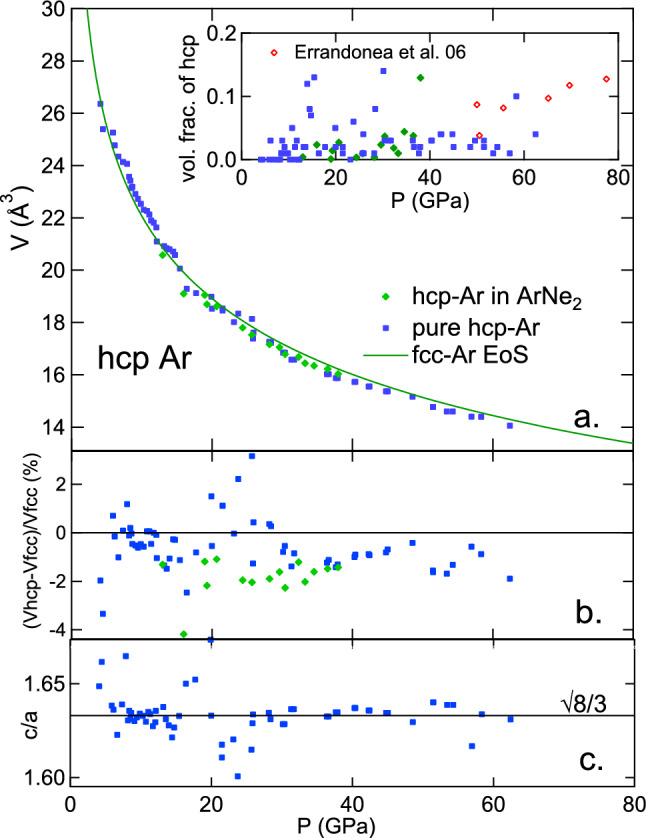
Equation of state of hcp-Ar. (**a**) Atomic volume vs pressure measured with Au standard^28^, compared with fcc EoS (see Fig. 9). Inset: volumetric fraction of hcp-Ar in argon ($V_{hcp}/(V_{fcc}+V_{hcp})$) evaluated with a Rietvelt analysis of XRD spectra. (**b**) Relative difference between atomic volumes of fcc and hcp Ar measured on the same XRD spectrum. (**c**) *c*/*a* lattice parameters ratio; the horizontal line indicates the ideal ratio for hexagonal close packing.

The volume fraction of hcp Ar obtained by analysis of XRD data from runs 1 and 2 is represented in Fig. 10a. The scatter of ± 6% represents the uncertainty on this fraction. It mildly increases up to $\sim$ 4% at 61 GPa in both samples. This is in agreement with the measurements by Errandonea et al. after laser heating at 2500 K above 50 GPa^5^ (see Fig. 10a). Heating above room temperature thus does not favor the new phase, ruling out a kinetic control of the transformation in experiments, similarly to Kr and Xe fcc $\rightarrow$ hcp transition^11,12^. No hcp XRD signal was detected in an Ar sample compressed up to 248 GPa at 300 K^6^, which shows that hcp was still a minor phase at that pressure. A very wide fcc/hcp coexistence domain is thus expected for Ar at 300 K, extending from 4.6 GPa to pressures higher than 248 GPa, a three-fold increase in density. The pressure at which hcp phase becomes dominant increases with decreasing *Z* in rare-gas solids: 80 GPa for Xe^12^, $\sim$ 400 GPa for Kr^11^, and the hcp phase has not been observed in Ne and He up to $\sim$ 200 GPa^23,34^. Fcc and hcp phases of argon coexist in a very wide pressure domain; this is in line with theoretical description of this rare gas solid, which finds that the free energy of the two close-packed phases is close and evolves similarly with compression^7^.

This work reveals that argon fcc $\rightarrow$ hcp transformation is facilitated by non-hydrostatic stress: hcp phase is observed at lower pressure in argon samples directly compressed between the anvils (runs 1 and 4) than in argon compressed in a softer pressure medium (runs 2 and 3). Similarly, a transformation to an hcp phase has been observed in cryogenic Ar-1%N$_2$ mixtures, but only after cold-working^35^; Barrett and Meyer conclude that plastic strain is necessary to induce stacking faults which will eventually produce the hcp modification, made stable by N$_2$ impurities. This is analogous to the behaviour of cobalt alloys, where metastable fcc phase transforms to hcp under plastic deformation, adopting a martensitic transformation mechanism^36^. It is a common view in metallurgy that defects associated to plastic strain (dislocations, stacking faults, twins) act as nucleation sites which can allow the system to adopt the lattice with the lowest energy. However, in the case of pressurized argon the amount of hcp phase remains small up to 114 GPa, even under high plastic strain, raising the question of hcp vs fcc thermodynamic stability. We cannot conclude whether plastic strain and associated stacking faults facilitates a transformation to a stable phase, or creates minor amounts of an unstable phase. Recent works indicate that stacking faults may result in the synthesis of unstable phases under high compression. Gold and silver, when compressed by shock waves to above 150 GPa, transform from a fcc to a body-centered cubic phase, which is not observed after static compression to the same conditions. It has been suggested that stacking faults, which are massively created by the shock uniaxial stress, promote this structural transformation^37^. Even if here, the formation of stacking faults in argon is probably not related to uniaxial stress, as discussed in Ref.^12^, they might favor hcp formation.

The hcp *c*/*a* ratio in hcp Ar does not vary with pressure in run 1 and has an average value of 1.632, very close to the ideal $\sqrt{(}8/3)$ value (see Fig. 10c.). For run 2 data analysis, its value has been fixed to 1.63 because XRD signal did not allow a refinement of this parameter due to preferential orientation of grains. Similarly to Kr and Xe^11,12^, the measured volume for hcp Ar is slightly smaller than the volume of fcc Ar under the same conditions for most data points (see Fig. 10a,b), with an average difference of 1.0 %.

## Conclusion

In this work, the well controlled experimental conditions allowed to measure accurate equation of state parameters for argon in a wide compression range and at low to ambient temperature. We show that in this range, as expected for a rare gas solid^2,23^, fcc argon behaves like a quasi-harmonic solid; we have thus confidence that the Rydberg–Vinet and Mie–Gruneisen–Debye parameters measured here may be used out of the temperature range scanned in the current study (10–296 K) for pressure measurement purpose. The onset of a fcc to hcp phase transformation is detected already at 4.2 GPa, lower than reported before^5^. However, the amount of hcp remains small up to the maximum pressure reached here, 114 GPa, suggesting that the energy difference between fcc and hcp phases does not evolve significantly in a twofold compression range. The transition proceeds through stacking faults, and is facilitated by non-hydrostatic compression.

## Methods

Several samples have been loaded (see Table 1). Runs 2 and 3 samples were Ar–Ne mixtures, prepared, left one day to homogeneize and subsequently loaded, using a 1200 b compressor. The concentrations were calculated from the partial pressure of the gases, using equations of state including a Virial correction. Pure argon has been loaded in a compressor (runs 1 and 4) or cryogenically (run 5). In run 5, argon has been loaded with thin Pt foils and laser-heated prior to cryogenic experiment in order to release stress and produce smaller crystallites. They have been characterized up to 114 GPa with X-ray diffraction (XRD) on different platforms (see Table 1). The temperature in the experiment room was controlled to 296 K in runs 1–4; in run 5, a helium cryostat dedicated to diamond anvil cell experiments was used, with the temperature measured with a thermocouple.

Angular-dispersive XRD technique has been used, with an X-ray wavelength of 0.3738 Å; portions of XRD images are presented in Figs. 1 and 3. The X-ray spot size on the sample was 10 $\times$ 15 $\upmu$m FWHM for runs 1–3, and 2.5 $\times$ 3 $\upmu$m for runs 4 and 5. The sample to detector distance was calibrated using a reference sample. The diamond anvil cell was rotated by ± 10$^{\circ }$ or ± 12$^{\circ }$ during XRD exposures for runs 1–4. The pressure was measured using a small sphere/foil of gold as an X-ray pressure calibrant^28^, or a ruby sphere with pressure effect from Ref.^38^ and temperature effect from Ref.^39^. In runs 2 and 3, the part of the mixture samples analyzed with XRD was chosen with the help of the on-line sample imaging setup.

**Table 1:** Conditions of the experiments.

Run	Platform	Sample composition	Culet diameter ($\upmu$m)	*P* range (GPa)	*T* range (K)	*P* gauge	*P* onset of hcp (GPa)
1	Psiche	100% Ar	300	1.4–62	296	Au^28^	4.2
2	Psiche	59% Ar-41% Ne	300	8–65	296	Au^28^	13
3	Psiche	40% Ar-60% Ne	300	4–61	296	Au^28^	35
4	ID27	100% Ar	100 $\times$ 300	1.6–114	296	Au^28^	10
5	ID27	100% Ar	300	1–25	10–296	ruby^38^	–

Psiche and and ID27 beamlines are located at Synchrotron Soleil and at European Synchrotron Radiation Facility, France, respectively. Diamond anvils culet diameter is indicated.

**Table 2:** Parameters of Mie–Grüneisen–Debye quasi-hydrostatic equation of state for fcc argon.

$V_0$	38.0 Å$^3$/at
$K_0$	2.65 GPa
$K'_0$	7.423
$\theta _{D0}$	93.3 K
$\gamma _0$	2.7

Subscript 0 indicates reference state, at ambient pressure and 0 K.

## Supplementary Information

Supplementary Information 1.
