# Supplementary material for: Stability and equation of state of face-centered cubic and hexagonal close packed phases of argon under pressure
Source: Sci Rep. 2021 Jul 26;11:15192. doi: 10.1038/s41598-021-93995-y (PMC8313556; doi:10.1038/s41598-021-93995-y)
Supplement: Supplementary file 1 — Supplementary Information 1. [file 41598_2021_93995_MOESM1_ESM.pdf]

Supplementary materials for

Stability and equation of state of face-centered cubic and hexagonal close packed phases of argon under pressure

Agnès Dewaele[1,2], Angelika D. Rosa[3], Nicolas Guignot[4], Denis Andrault[5], João Elias F.S. Rodrigues3, and Gaston Garbarino3

[1]CEA, DAM, DIF, F-91297 Arpajon, France

[2]Université Paris-Saclay, CEA, Laboratoire Matière en Conditions Extrêmes, 91680 Bruyères-le-Châtel, France

[3]ESRF, BP220, F-38043, France

[4]Synchrotron Soleil,F-91192 Saint Aubin, France

[5]Universit é Clermont Auvergne, CNRS, IRD, OPGC, LMV, Clermont-Ferrand, France

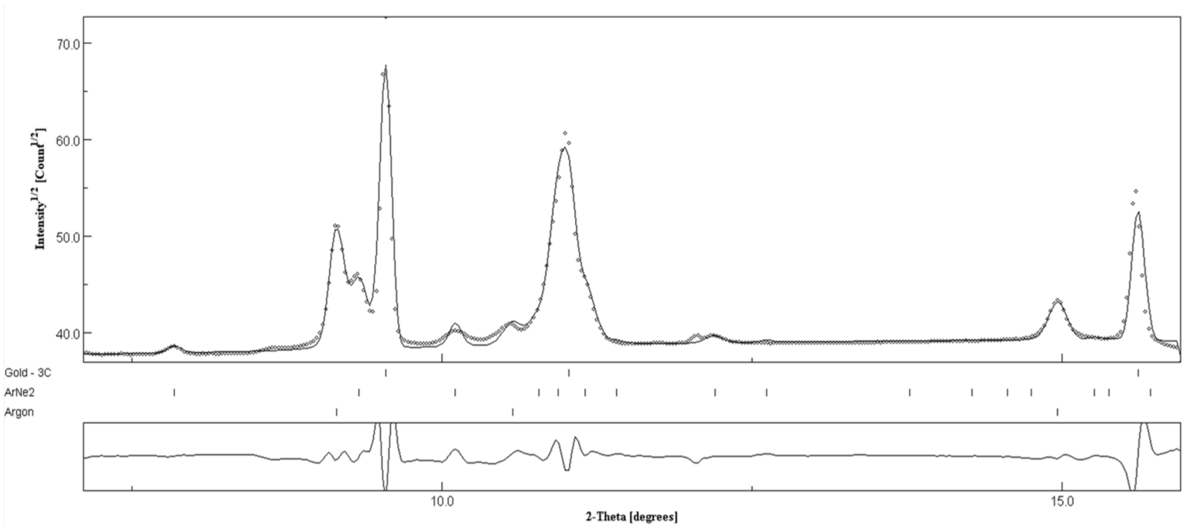

Fig. S1: Rieltvelt refinement of an X-ray diffraction spectrum collected at 36.5 GPa in run 2. Gold, $ArNe_2$ and argon volumetric fraction, lattice parameters and preferred orientations have been refined.

Ar Fcc EoS data points

RUN 1:

aAr(angstrom)	aAu(angstrom)	PAu (GPa)
5.0865998	4.0668998	1.3463738
5.0862002	4.0668998	1.3463738
5.0862002	4.0668998	1.3463738
5.0861001	4.0665998	1.3850833
5.0847998	4.0668998	1.3463738
5.0766001	4.0665998	1.3850833
5.0535002	4.0655999	1.5145164
5.0448999	4.0644999	1.657652
5.0314999	4.0640001	1.7228817
5.024200	4.0637999	1.7490647
5.0026999	4.0626998	1.8933702
4.973000	4.0618	2.0118871
4.9650998	4.0612001	2.091229
4.9523001	4.0605998	2.1707859
4.9457998	4.0602999	2.2106135
4.9359999	4.0598998	2.2638218
4.9212999	4.0594001	2.3304832
4.9131999	4.0588002	2.4106238
4.9036002	4.0574999	2.585155
4.8695002	4.0556002	2.8419452
4.8297000	4.0525999	3.2519403
4.7877998	4.0489001	3.7652061
4.7690001	4.0475001	3.9616601
4.7557001	4.0465002	4.1026778
4.7185998	4.0440001	4.4581442
4.6469998	4.0342002	5.8898664
4.6297998	4.0325999	6.1295948
4.6163001	4.0290999	6.6597977
4.5862999	4.0247998	7.3223815
4.5651002	4.0212002	7.8866816
4.5531998	4.0194001	8.1721964
4.5390000	4.0181999	8.3637552
4.5337000	4.0165	8.6368599
4.5244002	4.0172	8.524189
4.5166001	4.0137	9.0911226
4.505600	4.0116	9.4354
4.4910002	4.0091	9.8493299
4.4773998	4.0065999	10.26773
4.4650998	4.0039001	10.724707

4.4556999	4.0019002	11.066583
4.4482002	4.0004001	11.324975
4.4355001	3.9981999	11.706903
4.4246001	3.9962001	12.057191
4.4166999	3.9949	12.286502
4.4015999	3.9958	12.127607
4.3937998	3.9899001	13.180307
4.3899999	3.9879	13.543186
4.3808999	3.9856999	13.945948
4.3635001	3.983	14.445292
4.3544002	3.9814999	14.725177
4.3295002	3.9777999	15.423156
4.2929001	3.9728	16.383673
4.2564998	3.9660001	17.72258
4.2136002	3.9553001	19.907446
4.207900	3.9549999	19.970125
4.1855001	3.9477999	21.497356
4.1795998	3.9477999	21.497356
4.1616001	3.9403999	23.114733
4.155900	3.9375999	23.739559
4.1275001	3.9291	25.680477
4.1293998	3.9284	25.843334
4.1241002	3.9281001	25.913231
4.097600	3.9187	28.147892
4.0959001	3.9177001	28.390614
4.0798998	3.9107001	30.117319
4.0766001	3.9096999	30.36801
4.0662999	3.9059999	31.304182
4.0588999	3.9045	31.68766
4.0184002	3.8868999	36.361969
4.0169001	3.8859999	36.609863
4.0086002	3.8822999	37.638229
4.0071001	3.8814001	37.890587
3.990500	3.8729999	40.289982
3.9892001	3.8726001	40.406136
3.973700	3.8662	42.290833
3.9742999	3.8657999	42.410183
3.9575000	3.858	44.775215
3.9558001	3.8571999	45.021835
3.9300001	3.8429	49.561665
3.934200	3.846	48.556164
3.915000	3.8371999	51.442181
3.915900	3.8371	51.475574
3.901500	3.8313	53.432461
3.8966999	3.8288	54.289402

3.8694999	3.8211999	56.9454
3.8728001	3.8173001	58.33839
3.8554001	3.8062	62.417995
3.8457999	3.8064001	62.342968

RUN 2:

Name	aAr(angstrom)	aAu(angstrom)	aANe2(angstrom)	PAu (GPa)
ArNe12_009	4.5187998	4.0191998	5.0942001	8.3645124
ArNe12_011	4.4965	4.0173001	5.0743999	8.6697845
ArNe12_012	4.4822998	4.0148001	5.0668001	9.0753307
ArNe12_013	4.4432001	4.007	5.0250001	10.369095
ArNe12_014	4.4166002	4.0009999	4.9945998	11.394131
ArNe12_015	4.369	3.9921999	4.9510002	12.945745
ArNe12_016	4.3039999	3.9760001	4.8800001	15.957357
ArNe12_017	4.2858	4.8579998		
ArNe12_018	4.2561998	3.9609001	4.822	18.954237
ArNe12_019	4.244	3.9593999	4.8070002	19.262348
ArNe12_020	4.2224998	3.9526999	4.7855	20.661957
ArNe12_021	4.1718998	3.9358001	4.7164998	24.367817
ArNe12_022	4.1518002	3.9300001	4.7034001	25.699585
ArNe12_023	4.1206999	3.9195001	4.6719999	28.191395
ArNe12_024	4.1079001	3.9137001	4.6564002	29.613613
ArNe12_025	4.0956001	3.9105	4.6391001	30.412565
ArNe12_026	4.0723	3.9030001	4.6178002	32.32552
ArNe12_027	4.0641999	3.8994999	4.6046	33.238026
ArNe12_028	4.0503998	3.8945999	4.5918002	34.536922
ArNe12_030	4.0381999	3.8875	4.5728002	36.464207
ArNe12_031	4.0219002	3.8821001	4.5569	37.966576
ArNe12_032	4.0176001	3.8807001	4.5569	38.361313
ArNe12_034	3.9782	3.8654001	4.5159001	42.819221
ArNe12_035	3.9669001	3.85695	4.5023999	45.397552
ArNe12_036	3.9523001	3.8529999	4.4868999	46.632141
ArNe12_037	3.9335001	3.8441999	4.4651999	49.451134
ArNe12_038	3.9223001	3.8383999	4.4482999	51.362003
ArNe12_039	3.9107001	3.8327	4.4390998	53.28175
ArNe12_040	3.8954	3.8268001	4.4246998	55.313431
ArNe12_041	3.8813	3.8199999	4.3976998	57.712513
ArNe12_042	3.8694	3.8138001	4.3846998	59.954517
ArNe12_043	3.8591001	3.8089001	4.3771	61.76408
ArNe12_044	3.8522	3.8052001	4.369	63.152905
ArNe12_047	3.8439	3.8013999	4.3622999	64.599632

RUN 3:

Name aAr(angstrom) aAu(angstrom) PAu (GPa)

ArNe13_005-6	4.7275	4.0464001	4.2602453
ArNe13_009	4.7001	4.0436001	4.6604247
ArNe13_0011-12	4.6869998	4.0430002	4.7468047
ArNe13_0013-16	4.6427999	4.0381999	5.4464064
ArNe13_0018-19	4.6292	4.0352001	5.8911371
ArNe13_0020-21	4.5939002	4.0306001	6.5846238
ArNe13_0024	4.5914998	4.0306001	6.5846238
ArNe13_0025	4.5797	4.0293002	6.7831316
ArNe13_0026	4.5531998	4.0247998	7.4791861
ArNe13_0027	4.5093002	4.0181999	8.5248728
ArNe13_0028	4.4622002	4.0103002	9.8164101
ArNe13_0029	4.4137001	4.0012002	11.359509
ArNe13_0030	4.3555002	3.9886	13.597339
ArNe13_0031	4.3048	3.9751999	16.111469
ArNe13_0032	4.2887998	3.9700999	17.106014
ArNe13_0033	4.2670002	3.9646001	18.20244
ArNe13_0034	4.2256999	3.9532001	20.556126
ArNe13_0035	4.1918001	3.9425001	22.867933
ArNe13_0036	4.1704998	3.9366	24.186586
ArNe13_0037	4.165	3.9358001	24.367817
ArNe13_0038	4.1357999	3.9258001	26.683664
ArNe13_0039	4.1114001	3.9170001	28.800377
ArNe13_0040	4.0805998	3.9065001	31.425695
ArNe13_0041	4.0612001	3.8991001	33.343071
ArNe13_0042	4.0419002	3.8931	34.93959
ArNe13_0043	4.0209999	3.8845999	37.267117
ArNe13_0044	4.0039001	3.8778	39.185951
ArNe13_0045	3.9941001	3.8740001	40.280708
ArNe13_0046	3.9837	3.8701999	41.39193
ArNe13_0047	3.9595001	3.8571999	45.320099
ArNe13_0048	3.9417	3.8492	47.837669
ArNe13_0049	3.9236	3.8396001	50.963081
ArNe13_0052+53	3.8912001	3.8239	56.329006
ArNe13_0055+56	3.8717	3.8174	58.646305
ArNe13_0057+58	3.8606999	3.8104999	61.169586

RUN 4:

Name	aAr(angstrom)	aAu(angstrom)	PAu (GPa)
CDMX_19_060	4.9172921	4.0596285	2.4355764
CDMX_19_073	4.8566704	4.0561662	2.9027729
CDMX_19_078	4.6401639	4.0370808	5.6116343
CDMX_19_0113	4.5500975	4.0267358	7.1780849
CDMX_19_0120	4.5033321	4.0198112	8.2668238
CDMX_19_0126	4.4652271	4.0128865	9.3887148
CDMX_19_0127	4.3994088	4.0007682	11.434262

CDMX_19_0137	4.3543758	3.990381	13.273728
CDMX_19_0147	4.3301272	3.9851873	14.224258
CDMX_19_0151	4.3180027	3.981725	14.869564
CDMX_19_0157	4.2972178	3.9765313	15.855319
CDMX_19_0165	4.2747011	3.9713376	16.862717
CDMX_19_0174	4.2487206	3.9644132	18.240137
CDMX_19_0182	4.2123475	3.954026	20.38187
CDMX_19_0190-1	4.1972766	3.9489179	21.469194
CDMX_19_0192-3	4.1965394	3.9486024	21.537086
CDMX_19_0194-5	4.1955166	3.9481504	21.634537
CDMX_19_0196-7	4.1939459	3.9478476	21.699913
CDMX_19_0198_9	4.1925111	3.9472988	21.818621
CDMX_19_0200-1	4.1913438	3.9469497	21.894253
CDMX_19_0202-3	4.1890426	3.9463165	22.031744
CDMX_19_0204-5	4.1868057	3.9455855	22.190903
CDMX_19_0206-7	4.1853924	3.9452388	22.266546
CDMX_19_0208-9	4.1835165	3.9445872	22.409021
CDMX_19_0210-1	4.181993	3.9442239	22.488634
CDMX_19_0212-3	4.1795678	3.9434178	22.665678
CDMX_19_0214-5	4.1749644	3.9422083	22.932419
CDMX_19_0216-7	4.1721172	3.9414196	23.107065
CDMX_19_0218-9	4.1670103	3.9398715	23.4515
CDMX_19_0220-1	4.1622691	3.9384434	23.771183
CDMX_19_0222-3	4.1427898	3.9323514	25.155916
CDMX_19_0227-8	4.0941477	3.9164486	28.935532
CDMX_19_0229-30	4.080193	3.9107008	30.362143
CDMX_19_0231-2	4.0685792	3.9066303	31.39246
CDMX_19_0233-4	4.0585837	3.9028635	32.360901
CDMX_19_0235-6	4.0470977	3.8980486	33.62011
CDMX_19_0237-8	4.0380764	3.8948081	34.481247
CDMX_19_0240-41	4.0104895	3.8850152	37.151562
CDMX_19_0243-4	3.9922392	3.8763771	39.593971
CDMX_19_0245-6	3.9614098	3.8636718	43.339703
CDMX_19_0247-8	3.9499867	3.8582745	44.987839
CDMX_19_0249-50	3.9256899	3.8491445	47.855427
CDMX_19_0251-2	3.8802278	3.8293405	54.433022
CDMX_19_0253-4	3.8553214	3.8140862	59.849884
CDMX_19_0255-6	3.8498175	3.8114257	60.827194
CDMX_19_0257-8	3.8306706	3.8029191	64.018776
CDMX_19_0259-60	3.8148458	3.7937276	67.58432
CDMX_19_0261-2	3.7975237	3.785497	70.883247
CDMX_19_0263-4	3.787349	3.7788622	73.617416
CDMX_19_0265-6	3.7687504	3.769079	77.774483
CDMX_19_0267-8	3.7610307	3.7636943	80.127808
CDMX_19_0269-70	3.7466023	3.7570992	83.074829

CDMX_19_0271-2	3.7356997	3.7495911	86.518341
CDMX_19_0277-6	3.7351942	3.7478735	87.319572
CDMX_19_0278-9	3.7265444	3.744415	88.94838
CDMX_19_0280-1	3.7205105	3.7403214	90.903252
CDMX_19_0282-3	3.7113869	3.7356708	93.160011
CDMX_19_0284-5	3.6984558	3.727427	97.25605
CDMX_19_0286-7	3.6876848	3.7173266	102.44547
CDMX_19_0292-3	3.6709371	3.7069592	107.97408
CDMX_19_0294-5	3.6597521	3.7019014	110.74759
CDMX_19_0296-7	3.6497409	3.6965573	113.73376

RUN 5:

Name	aAr(angstrom)	Pruby (GPa)	T (K)
Ar_cell1_007	4.3660002		300
Ar_cell1_008	4.3119998	15.90158	280
Ar_cell1_009	4.2989998	16.642603	260
Ar_cell1_010	4.2979999	16.446001	240
Ar_cell1_011	4.2969999	16.513533	220
Ar_cell1_012	4.2979999		200
Ar_cell1_013	4.2979999	16.625416	180
Ar_cell1_014	4.2989998	16.549814	160
Ar_cell1_015	4.296	16.470644	140
Ar_cell1_016	4.2950001	16.531586	120
Ar_cell1_017	4.2950001	16.759277	100
Ar_cell1_018	4.2940001	16.773706	80
Ar_cell1_019	4.2919998	16.724966	60
Ar_cell1_020	4.2950001	16.75024	60
Ar_cell1_021	4.2940001	16.747358	40
Ar_cell1_022	4.2919998		40
Ar_cell1_023	4.3080001	15.422741	20
Ar_cell1_024	4.3610001	12.397645	10
Ar_cell1_025	4.4400001	9.5808287	5.5
Ar_cell1_026	4.3109999	15.758632	60
Ar_cell1_027	4.322	15.00287	120
Ar_cell1_028	4.3280001	14.777557	200
Ar_cell4_002	4.9759998		300
Ar_cell4_012	4.9029999	2.067589	250
Ar_cell4_013	4.8629999	2.3094366	200
Ar_cell4_014	4.8049998	2.7639737	152
Ar_cell4_015	4.6929998	4.1876454	100
Ar_cell4_016	4.6659999	4.5802059	81
Ar_cell4_017	4.6490002	4.8110256	60
Ar_cell4_018	4.6479998	4.9496875	40
Ar_cell4_020	4.6479998	4.8734231	20
Ar_cell4_021	4.6490002		10

Ar_cell4_024	4.645	10	
Ar_cell4_026	4.79	300	
Ar_cell4_027	4.7690001	3.3717213	300
Ar_cell4_027	4.7690001	3.3717213	300
Ar_cell4_029	4.6999998	4.2439122	250
Ar_cell4_030	4.6799998	4.2702632	200
Ar_cell4_031	4.678	4.3108034	150
Ar_cell4_032	4.6690001	4.3709726	100
Ar_cell4_033	4.664	4.4365506	80
Ar_cell4_034	4.6589999	4.5623713	60
Ar_cell4_035	4.6570001	4.6316872	40
Ar_cell4_036	4.6570001	4.6401515	20
Ar_cell4_037	4.6570001	4.5738025	10
Ar_cell5_011	5.0549998	1.1456162	250
Ar_cell5_012	5.0469999	1.0828884	200
Ar_cell5_013	4.9099998	1.9020382	150
Ar_cell5_014	4.9099998	1.9020382	150
Ar_cell5_016	4.9070001	1.8704457	100
Ar_cell5_017	4.9029999	1.841736	80
Ar_cell5_020	4.901	1.7804763	60
Ar_cell5_021	4.9000001	1.783337	40
Ar_cell5_023	4.901	1.7456526	20
Ar_cell5_024	4.9029999	1.698257	10
Ar_cell5_025	4.454	10.139159	300
Ar_cell5_027	4.4229999	11.164268	300
Ar_cell5_027	4.4229999	11.164268	300
Ar_cell5_029	4.4180002	11.425138	250
Ar_cell5_030	4.4169998	11.439003	200
Ar_cell5_031	4.4070001	11.774152	150
Ar_cell5_032	4.381	12.796848	100
Ar_cell5_033	4.3769999	12.844582	80
Ar_cell5_034	4.3759999	12.847823	60
Ar_cell5_036	4.3759999	12.928313	40
Ar_cell5_037	4.382	12.204872	20
Ar_cell5_038	4.395	11.451547	10
Ar_cell5_040	4.2649999	17.596375	300
Ar_cell5_043	4.23	19.665363	300
